# Supplementation of transport and freezing media with anti-apoptotic drugs improves ovarian cortex survival

**DOI:** 10.1186/s13048-016-0216-0

**Published:** 2016-02-12

**Authors:** Laurie Henry, Maïté Fransolet, Soraya Labied, Silvia Blacher, Marie-Caroline Masereel, Jean-Michel Foidart, Agnès Noel, Michelle Nisolle, Carine Munaut

**Affiliations:** Laboratory of Tumor and Development Biology, Groupe Interdisciplinaire de Génoprotéomique Appliquée (GIGA-R), Université de Liège, Tour de Pathologie (B23) Sart-Tilman, B-4000 Liège, Belgium; Department of Gynecology, University of Liège, Boulevard du XIIème de Ligne, B-4000 Liège, Belgium

**Keywords:** Ovarian cryopreservation, anti-apoptotic drugs, slow-freezing, fertility preservation

## Abstract

**Background:**

Ovarian tissue preservation is proposed to patients at risk of premature ovarian failure, but this procedure still needs to be optimized. To limit injury during ovarian tissue cryopreservation, anti-apoptotic drugs were added to the transport and freezing media of ovarian cortex tissue.

**Methods:**

Sheep ovaries were transported, prepared and frozen in solutions containing vehicle or anti-apoptotic drugs (Z-VAD-FMK, a pan-caspase inhibitor, or sphingosine-1-phosphate (S1P), a bioactive lipid). After the tissue was thawed, the ovarian cortex was cultured for 2 or 6 days. Follicular quantification and morphological and proliferation analyses were performed on histological sections.

**Results:**

After 2 days of culture, S1P improved the quality of primordial follicles; higher densities of morphologically normal and proliferative primordial follicles were found. Z-VAD-FMK displayed similar effects by preserving global primordial follicular density, but this effect was evident after 6 days of culture. This drug also improved cell proliferation after 2 and 6 days of culture.

**Conclusions:**

Our results showed that the addition of S1P or Z-VAD-FMK to the transport and freezing media prior to ovarian tissue cryopreservation improves primordial follicular quality and therefore improves global tissue survival. This should ultimately lead to improved fertility restoration after auto-transplantation.

**Electronic supplementary material:**

The online version of this article (doi:10.1186/s13048-016-0216-0) contains supplementary material, which is available to authorized users.

## Background

Currently, patients with cancer clearly benefit from aggressive chemotherapy, radiotherapy and bone marrow transplantation. However, these treatments can induce premature ovarian failure (POF) in girls or young women. Ovarian cryopreservation should be proposed to these patients before they begin treatment. This procedure has already allowed the birth of more than 40 babies across the world [1–3], but it still needs to be optimized. Indeed, ovarian cortex cryopreservation followed by auto-transplantation after cancer remission affects the quality of the ovarian tissue. The primordial follicular reserve decreases, which consequently impairs the survival lifespan of the grafted tissue fragments, limiting the possibility for procreation.

Ovarian transplantation is avascular, which results in tissue ischemia over the 5 first days after grafting and is sometimes associated with reperfusion injury [4, 5]. This transient hypoxic period is followed by gradual oxygenation, leading to reperfusion of the ovarian transplant. This process represents the main origin of ischemia and follicular loss [6, 7]. The freezing procedure also induces tissue damage. In fact, slow freezing is the gold standard for ovarian tissue cryopreservation. However, this technique is known to lead to tissue fibrosis [8] and to alter the viability of both stromal and follicular cells within the ovarian samples [9, 10]. Apoptosis also plays an important role in cryo-injuries, mainly by the activation caspases and the Fas system [11–13]. Indeed, caspase activation was observed in frozen-thawed tissue with preserved architecture [14].

Several anti-apoptotic drugs have already been proposed to improve fertility preservation. They were studied along with preventive treatment during oncologic therapy rather than during the ovarian tissue cryopreservation process, including during freezing or transplantation. Among these drugs, imatinib, a kinase inhibitor that mainly targets the ABL family members KIT and PDGFR, protects ovarian tissue during chemotherapy by inhibiting cisplatin-induced Tap63-α phosphorylation [15]. Sphingosine-1-phosphate (S1P) is a bioactive lipid in follicular fluid that has two interesting properties: it is anti-apoptotic and pro-angiogenic [16]. In several studies, S1P has been shown to protect the follicle reserve by decreasing apoptosis during chemotherapy and radiotherapy [17–22].

On the other hand, anti-apoptotic drugs have been used during the cryopreservation process to prevent the activation of apoptotic pathways and subsequently improve tissue survival during freezing and grafting. The addition of Z-VAD-FMK, a pan-caspase inhibitor, to the freezing and thawing media has been shown to decrease the apoptosis rate and the number of days before the estrous cycle is resumed following auto-transplantation of cryopreserved mouse ovaries [23]. S1P has also been used in the vitrification media before the transplantation of mouse ovaries and conferred significant protection of primordial follicles after grafting [24, 25].

Anti-apoptotic drugs have also been evaluated during tissue transplantation. In an auto-transplantation model of fresh sheep ovarian fragments into the abdominal wall, S1P did not show a beneficial effect [26]. However, the use of the same drug in xenografts of fresh human ovarian cortex in immunodeficient mice improved angiogenesis and decreased follicular apoptosis [27].

Meanwhile, these anti-apoptotic drugs could have a protective effect on ovaries during oncologic treatment or transplantation in mice, but the use of these anti-apoptotic drugs in vivo during oncologic treatment is only possible in animal models. However, their use in cryopreservation media alone appears to be safer and could be applicable to humans.

In vitro culture of thawed ovarian tissue is an important approach to use to analyze tissue after the cryopreservation process because the integrity of the tissue immediately after thawing may not reflect its true state [10, 28, 29]. It also enables observation of the effects of cryopreservation without transplantation.

The purpose of our study was to analyze and compare the effects of two anti-apoptotic drugs, Z-VAD-FMK and S1P, when added to the transport and freezing media for sheep ovarian cortex pieces, on survival during in vitro culture of the tissue immediately after thawing.

## Methods

### Ovarian tissue sampling

**Fig. 1:**
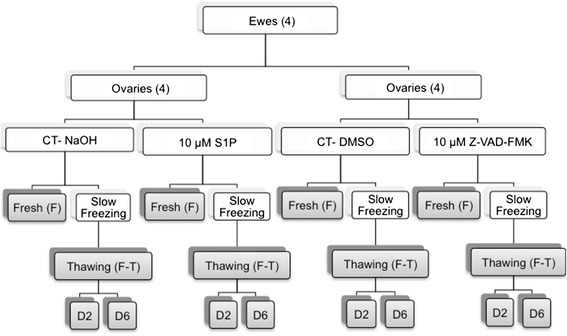
Study design describing the different conditions. Eight ovaries were cut into 2 parts and distributed among the four groups of transport media containing anti-apoptotic drugs (10 μM S1P or 10 μM Z-VAD-FMK) or vehicle (Control (CT); NaOH or DMSO). After the tissue was prepared, the ovarian cortex was cut into pieces with a biopsy punch to obtain similarly sized fragments (diameter of 2 mm). These pieces (24 punches per group), which were from different ovaries, were mixed and then randomly assigned to processing for the fresh histological analyses (6 punches) or to freezing (6 punches). After freezing and thawing, 6 punches were fixed (F-T), and the others were cultured for 2 or 6 days (D2 or D6). For histology, each entire punch was cut, and 12 sections were analyzed for follicular density and morphological assessment, whereas 3 sections were analyzed for proliferation

This study was approved by the Animal Ethics Committees of the Universities of Liège and Namur. Our experimental design is detailed in Fig. 1. Four ewes, 4 and 5 months old, were obtained from the Ovine Research Center of Namur University, and their ovaries were collected immediately after the ewes were euthanized. The ovaries were cut into 2 pieces, and one piece of each ovary was immersed in transport media composed of Leibovitz L-15 medium (Lonza, Verviers, Belgium, BE12-700 F) supplemented with 10 % normal sheep serum (Hormonology Laboratory, Belgium) and anti-apoptotic drugs. The anti-apoptotic drugs included 10 μM Z-VAD-FMK (R&D, United Kingdom, FMK001), which was diluted in DMSO, and 10 μM S1P (Biomol, Germany, Cay62570-10), which was diluted in 0.3 M NaOH. In the control groups, the anti-apoptotic drugs were replaced with the appropriate vehicles, namely, DMSO for Z-VAD-FMK and NaOH for S1P.

The ovaries were further prepared by removing medullary tissue, and the cortex was cut in 2-mm-diameter round pieces with a disposable biopsy punch (Miltex, Germany, 33-31-P/25). Twenty-four pieces per condition were obtained for the cryopreservation process, and 6 were immediately fixed in 4 % formaldehyde and embedded in paraffin for histological analysis (fresh condition, F). Throughout the entire process of ovarian cortex preparation, the ovaries were kept at 4 °C.

### Freezing and thawing of ovarian tissue

Cryopreservation was performed by slow freezing according to the technique first described by Gosden [30, 31]. Z-VAD-FMK (10 μM) or S1P (10 μM) was added to the cryopreservative medium. For thawing, cryovial tubes were removed from liquid nitrogen, left at room temperature for 2 min and then immersed in a 37 °C water bath at until the tissue was completely thawed. The ovarian pieces were subsequently washed three times in 37 °C culture medium without serum to remove the cryoprotective agents. Each wash lasted for 5 min. For each group, 6 pieces were fixed in 4 % formaldehyde and embedded in paraffin for histological analysis (frozen-thawed condition, F-T).

### In vitro culture of ovarian pieces

Thawed ovarian fragments were individually transferred to a Cellstar® 96-well plate U-Bottom (Greiner Bio-One, Italy, 650 185). Each well was filled with 100 μL of Dulbecco’s Modified Eagle Medium (Life Technologies, Belgium, 31053-028) supplemented with 200 mM L-Glutamine (Life Technologies, Belgium, 25030024), 100 μg/ml ascorbic acid, 1 μl/ml Insulin-Transferrin-Selenium solution (Life Technologies, Belgium, 41400-045), 1 μl/ml penicillin-streptomycin (10,000 U/mL; Life Technologies, Belgium, 15140-122), 25 mIU/ml recombinant FSH (Merck, Germany, Gonal-F®) and 5 % normal sheep serum. The tissue fragments were cultured for 2 (D2 condition) or 6 days (D6 condition) at 37 °C in a humidified incubator with 5 % CO_2_ and 5 % O_2_ as described by Sanfilippo [28]. The ovarian punches were fixed in 4 % formaldehyde, embedded in paraffin and cut into 5-μm serial sections for histological analysis.

### Histological analysis

Virtual images were acquired as previously described with an automatic digital slide scanner NanoZoomer 2.0HT (Hamamatsu, Belgium) [32].

To limit the effect of the heterogeneous distribution of the follicular pool within the ovarian cortex, 12 sections per ovarian piece, which covered the entire fragment, were analyzed as previously described [33]. Follicular quantification and morphological evaluation were performed as previously described [34]. Briefly, primordial follicles were considered degenerated if they contained disorganized granulosa cells, shrunken ooplasm or pyknotic oocytes.

Cell proliferation was evaluated by Ki-67 immunolabeling using MIB-1, a monoclonal mouse anti-human Ki-67 antigen clone (Dako, Denmark, M7240), at 1/100. Dako EnVision + HRP anti-mouse (Dako, Denmark, K4001) and DAB+ (K3468, Dako, Belgium) were used to view the immunolabeling.

Follicles were considered proliferative if at least one Ki-67-positive granulosa cell was observed at ×200 magnification (Leica ICC50 HD Camera, Belgium).

Stromal cell proliferation was automatically quantified using the image analysis toolbox of MATLAB 8.1.0.604 (R2013a) (MathWorks, Inc.). Because cell detection was mainly based on color segmentation, contrast was first enhanced by determining the excess of the red component (two times red value minus blue value minus green value). Then, based on the enhanced red component of the resulting color image, binary images of the cells (i.e., pixels belonging to cells were assigned an intensity of 1, whereas background pixels were assigned an intensity of 0) were obtained using an automatic entropy threshold [35]. To eliminate small artifacts, morphological filters [36] were applied on the resultant images. Binary images of the total tissue sections were obtained by applying an appropriate threshold to the blue component of the images. Lastly, cell density was defined as the area occupied by cells divided by the total area of the section.

### Statistical analyses

After the outcomes were logarithmically transformed, a linear mixed model was fit to the data to test for differences between the treatments and the number of days in culture. In this model, the ovarian fragment was introduced as a random factor. To correct for multiple comparisons and to avoid type I errors, the level of statistical significance was set at *p* = 0.01. Calculations were always carried out on the maximum amount of data available. The data analysis was carried out using the SAS (version 9.3 for Windows) statistical package.

## Results

### Follicular density

The investigation of follicular density in H&E stained sections revealed a very low number of primary and secondary follicles, which precluded statistical analysis. The analysis was therefore performed only on primordial follicles.

**Fig. 2:**
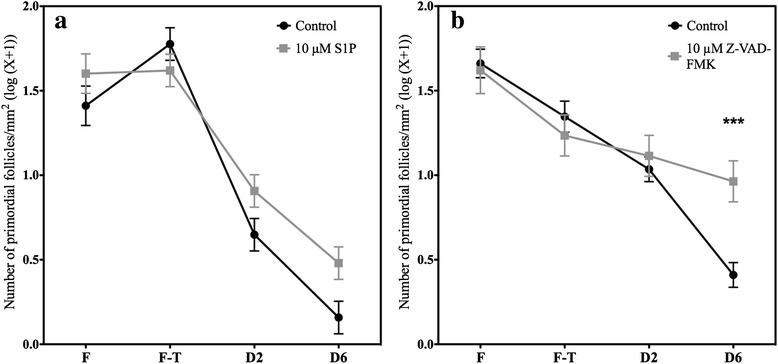
Analysis of total primordial follicle density. The number of primordial follicles (log(number / mm^2^ + 1)) in the ovarian fragments in the different conditions, fresh (F), frozen-thawed (F-T), and after 2 (D2) and 6 days (D6) of culture, was quantified and compared between the control groups and the **a** 10 μM S1P and **b** 10 μM Z-VAD-FMK groups. *** *p* < 0.0001

Evaluation of the primordial follicular density demonstrated that supplementation with S1P did not completely prevent primordial follicular loss during culture. Nevertheless, follicular density was higher in the S1P-treated group compared to the control group (Fig. 2b, Table 1 and Additional file 1: Tables S1 and S2).

**Table 1:** Primordial follicle density (number of primordial follicles/mm^2^ (log(X + 1))

	F	F-T	D2	D6
Control NaOH	1.4111	1.7764	0.6482^**^	0.1584^***^
10 μM S1P	1.6012	1.6200	0.9070^**^	0.4799^***^
Control DMSO	1.6609	1.3464^*^	1.0350^**^	0.4099^***^
10 μM Z-VAD-FMK	1.6209	1.2348^*^	1.1145	0.9636

^*^ *p* < 0.01 between the frozen-thawed (FT) and the fresh (F) tissue

^**^ *p* < 0.01 between tissue after 2 days of culture (D2) and frozen-thawed tissue

^***^ *p* < 0.01 between 6 (D6) and 2 days of culture

All *p* values are provided in Additional file 1: Tables S1 and S2

Z-VAD-FMK significantly decreased follicular loss after cryopreservation compared to vehicle (Fig. 2b). Indeed, post-thawing follicular density was maintained after up to 6 days of culture after Z-VAD-FMK treatment, while there was a constant decrease in the control group (Table 1 and Additional file 1: Table S1). Therefore, after 6 days of culture, more follicles were observed in the treated group than in the control group (Fig. 2a and Additional file 1: Table S2).

### Follicular quality

#### Morphology of primordial follicles

**Fig. 3:**
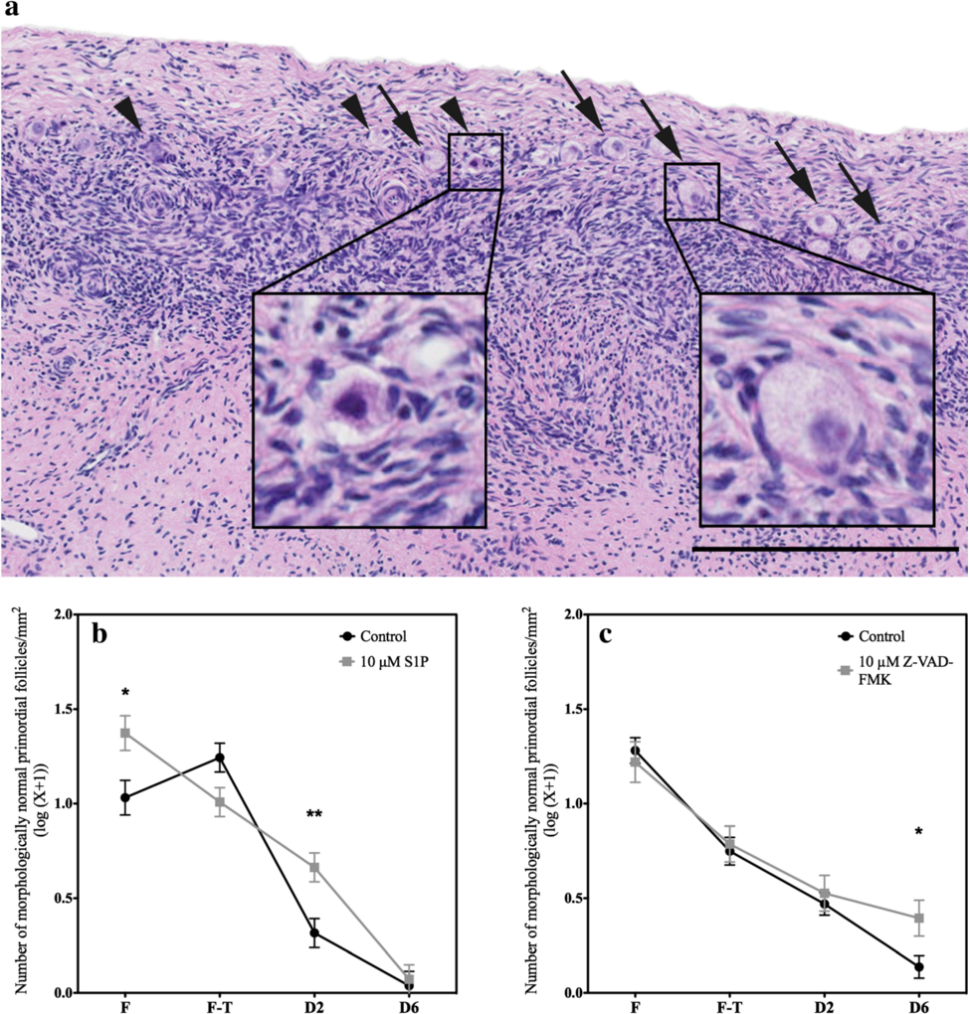
Analysis of primordial follicle morphology. **a** Illustration of primordial follicles considered morphologically normal (*arrows*) or abnormal (*arrowheads*). Abnormal primordial follicles were characterized by disorganized granulosa cells, shrunken ooplasm and/or pyknotic oocytes. The density of morphologically normal primordial follicles (log(number / mm^2^ + 1)) in the ovarian fragments under the different conditions, fresh (F), frozen-thawed (F-T), and after 2 (D2) and 6 days (D6) of culture, was quantified and compared between the control group and the **b** 10 μM S1P and **c** 10 μM Z-VAD-FMK groups. Scale bar: 250 μm. * *p* < 0.01. *** *p* < 0.0001

Primordial follicle morphology was evaluated in H&E sections (Fig. 3a). Morphologically normal follicles were distinguished from degenerated follicles. This analysis showed that the freezing process decreased the density of morphologically normal primordial follicles. This reduction continued progressively throughout the culture duration.

Supplementation with S1P preserved follicular morphology; compared to the density of normal follicles found in the control tissues, the density of normal follicles was greater in the fresh tissue and after 2 days of culture, whereas this difference faded after 6 days in culture (Fig. 3b, Table 2 and Additional file 1: Tables S3 and S4).

**Table 2:** Morphologically normal primordial follicle density (number of primordial follicles/mm^2^ (log(X + 1))

	F	F-T	D2	D6
Control NaOH	1.0316	1.2435	0.3161^**^	0.03793^***^
10 μM S1P	1.3735	1.0081^*^	0.6635^**^	0.07230^***^
Control DMSO	1.2810	0.7495^*^	0.4699^**^	0.1374^***^
10 μM Z-VAD-FMK	1.2200	0.7870^*^	0.5263^**^	0.3946

^*^ *p* < 0.01 between the frozen-thawed (FT) and the fresh (F) tissue

^**^ *p* < 0.01 between tissue after 2 days of culture (D2) and frozen-thawed tissue

^***^ *p* < 0.01 between 6 (D6) and 2 days of culture

All *p* values are provided in Additional file 1: Tables S3 and S4

Compared to the effects of S1P, the effects of Z-VAD-FMK seemed to occur later. Compared to vehicle, Z-VAD-FMK significantly preserved the density of morphologically normal follicles between 2 and 6 days of culture (Fig. 3c, Table 2 and Additional file 1: Table S3). Therefore, the density of normal primordial follicles that was observed after 6 days of culture was higher in the treated group than in the control group (Fig. 3c and Additional file 1: Table S4).

#### Granulosa cell proliferation

**Fig. 4:**
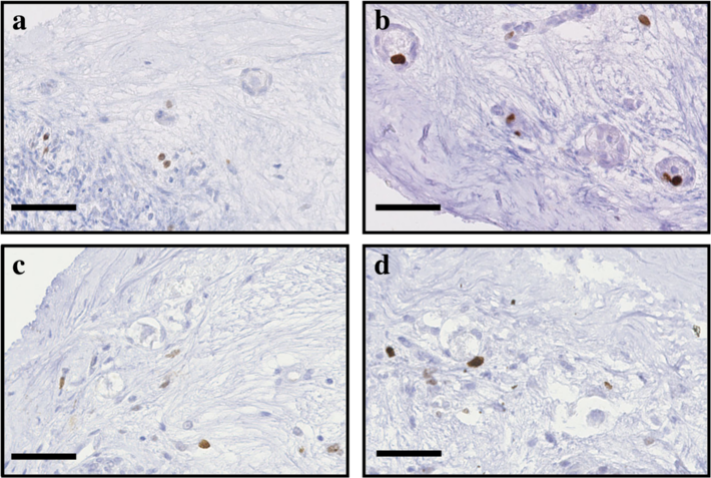
Illustration of proliferative cells when treated with S1P. Proliferative granulosa and stromal cells, immunostained with a Ki67 AB, in ovarian fragment after 2 and 6 days of culture in the NaOH control group (respectively **a** and **c**) and the 10 μM S1P group (respectively **b** and **d**). Scale bar: 50 μm

**Fig. 5:**
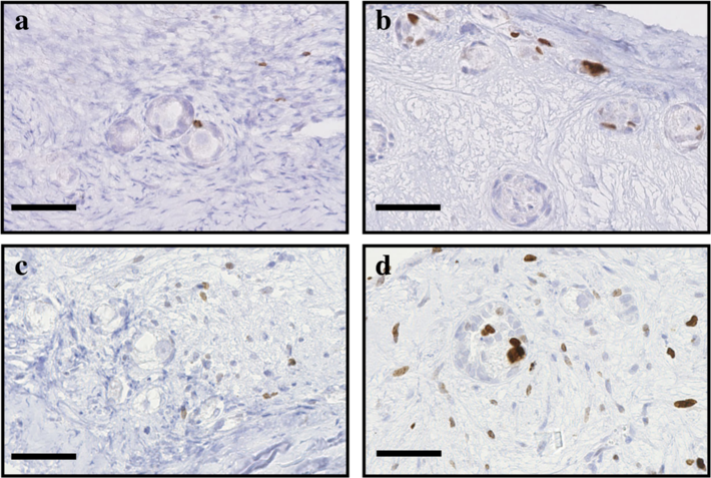
Illustration of proliferative cells when treated with Z-VAD-FMK. Proliferative granulosa and stromal cells, immunostained with a Ki67 AB, in ovarian fragment after 2 and 6 days of culture in the DMSO control group (respectively **a** and **c**) and the 10 μM Z-VAD-FMK group (respectively **b** and **d**). Scale bar: 50 μm

**Fig. 6:**
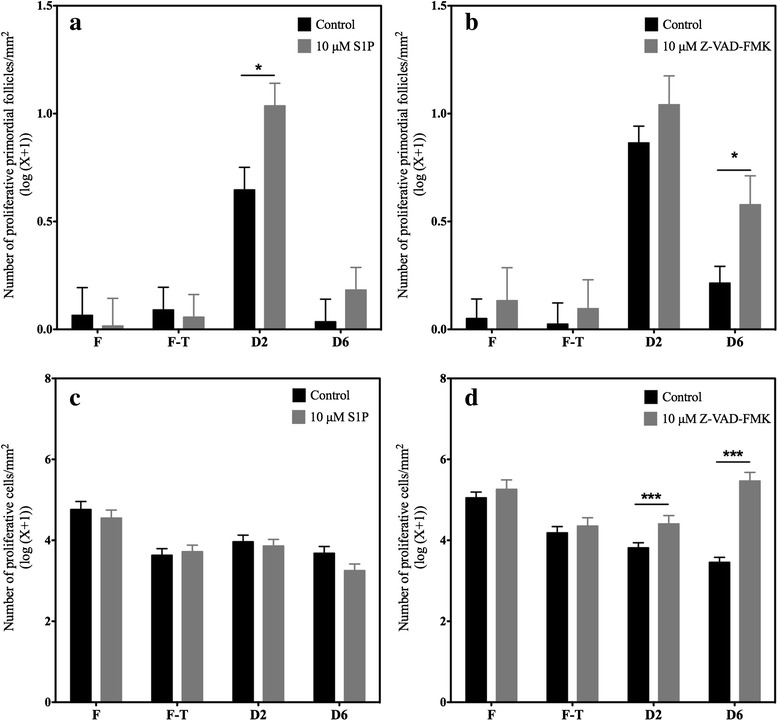
Analysis of proliferative cells in the ovarian fragments. Primordial follicles containing proliferative granulosa cells (log(number/mm^2^ + 1)) in the ovarian fragments under the different conditions, fresh (F), frozen-thawed (F-T), and after 2 (D2) and 6 days (D6) of culture, were quantified and compared between the control groups and the **a** 10 μM S1P and **b** 10 μM Z-VAD-FMK groups. All proliferative cells within the whole ovarian fragment (log(number/mm^2^ + 1)) were quantified for the ovarian fragments treated with **c** 10 μM S1P or **d** 10 μM Z-VAD-FMK. Scale bar: 250 μm. * *p* < 0.01. *** *p* < 0.0001

Granulosa cell proliferation, as analyzed by Ki-67 immunostaining, was used as a second marker of follicular health (Figs. 4 and 5). Granulosa cells that were associated with healthy follicles were able to proliferate as soon as 2 days of culture; thereafter, proliferation seemed to decrease, as observed after 6 days (Fig. 6a and b). Supplementation with anti-apoptotic drugs resulted in a higher density of proliferative granulosa cells (which were defined as proliferative primordial follicles) after 2 days of culture with S1P (Figs. 4 and 6a and Additional file 1: Table S5) and after 6 days of culture with Z-VAD-FMK (Figs. 5 and 6b and Additional file 1: Table S5), confirming the beneficial effect of the 2 anti-apoptotic drugs on follicular health.

### Cell proliferation

Global proliferation (granulosa and stroma) in ovarian punches, as revealed by Ki-67 immunostaining was used to evaluate the quality of the ovarian tissue. The proliferation of follicles together with the presence of mitosis in granulosa cells serves as an indication of the viability of follicles that are grown in culture. These measures also indicate that frozen/thawed follicles can remain functional over the culture period.

Supplementation with S1P did not affect proliferation (Fig. 6c and Additional file 1: Table S6), whereas increased proliferative cell density was found after 2 and 6 days of culture after supplementation with Z-VAD-FMK compared to vehicle treatment, demonstrating that this anti-apoptotic drug improved tissue survival (Fig. 6d, Additional file 1: Table S6).

## Discussion

In the present study, ovarian tissues treated with anti-apoptotic drugs before and during cryopreservation showed significantly higher primordial follicle density and quality after 2 or 6 days of culture. Two different drugs, Z-VAD-FMK and SIP, were added in the transport and freezing media used for the sheep ovarian tissue. For SIP, these positive effects were evident over the short term, after 2 days in culture, whereas for Z-VAD-FMK, they were evident over the long term, after 6 days in culture. Our results demonstrated that Z-VAD-FMK supplementation during transport and slow freezing improved the survival of the ovarian tissue, as demonstrated by a higher rate of cell proliferation after 2 and 6 days of culture compared to that of the control. In our study, primary and more mature follicles were not analyzed due to their low numbers, and transitional follicles were considered primordial. Only immature follicles enable sustainable restoration of ovarian function after transplantation because antral follicles are not cryoresistant [2].

Ovarian tissue cryopreservation followed by auto-transplantation is a promising method that has produced good results. However, this technique still needs to be improved, including both the freezing and transplantation processes. Indeed, significant apoptotic loss of primordial follicles occurs during cryopreservation [13, 37]. In humans, the use of anti-apoptotic drugs within the site of transplantation is technically difficult and also raises ethical issues. We therefore decided to treat ovarian samples only ex vivo. Our aim was to assess if supplementation of the transport, preparation and cryopreservation media before transplantation of the ovarian cortex could be beneficial. The in vitro tissue culture system that was utilized was previously used by others to test the effects of drugs on ovarian tissue survival [10, 28, 29] and allowed us to evaluate tissue and follicle viability and integrity. Indeed, the analysis of cortex samples immediately after thawing may not reflect their real state [10, 28]. We therefore performed tissue culture for up to 6 days after the tissue was thawed.

The potential benefit of anti-apoptotic drugs such as Z-VAD-FMK was based on previous studies that described significant apoptotic loss of primordial follicles during slow freezing-based cryopreservation [13, 37] and by studies that described the role of caspases in the activation of apoptosis in granulosa cells via activation of the Fas system [12, 14]. This anti-apoptotic drug was already identified as a cryoprotective agent that improves the recovery and the survival of cryopreserved cells and vitrified porcine embryos [11, 38]. The bioactive lipid, S1P, has been demonstrated to limit ovarian toxicity during both chemo- and radiotherapy [17–21] (see Additional file: 2 FigureS1) . Addition of S1P in granulosa cells culture has shown to prevent apoptosis induced by oxidative stress [39]. Its protective effects during ovarian tissue cryopreservation and transplantation are more controversial. When used during auto-transplantation of fresh ovine tissue, no beneficial effect was observed [26]. The same effect was described when SIP was added to the freezing media of whole ovine ovaries [40] or to the culture media of ovarian tissue after slow freezing or vitrification [41]. S1P supplementation during vitrification of mouse ovaries improved the number of morphologically intact primordial follicles present after grafting [24, 25]. In our study, S1P did not preserve follicle density after cryopreservation but did improve follicle morphology after transport (fresh tissue) and after 2 days in culture, when increased granulosa cell proliferation was detected. We did not observe an S1P-mediated improvement in cell proliferation. However, S1P has already been shown to improve stromal cell proliferation, although these effects were observed for a higher concentration of S1P and after a different supplementation method [27].

## Conclusions

Our results showed that the addition of S1P or Z-VAD-FMK to the transport and freezing media prior to ovarian tissue cryopreservation improved primordial follicular quality and therefore improved global tissue survival.

However, the beneficial effects of these two compounds over 2–6 days in culture do not necessarily imply that this approach will improve engraftment and/or maintenance of primordial follicle health after transplantation. Eventually, it will be necessary to show that this approach improves the potential for conception by demonstrating normal follicular development. Similarly, improved oocyte health and fertilization will need to be demonstrated via healthy, live-born pregnancies and other supporting in vivo experimental evidence. Thus, at present, this model only provides a surrogate marker to encourage additional work in this area.

## Additional files

Additional file 1:
**Statistical analyses between the different conditions and treatments**. **Table S1** and **Table S2**. Primordial follicular density. **Table S3** and **Table S4**. Density of morphologically normal primordial follicules. **Table S5.** Density of proliferative granulosa cells between treatments. **Table S6.** Density of global proliferative cells between treatments. (XLSX 38 kb) Additional file 2: Figure S1. Cellular anti-apoptotic mechanisms of S1P and Z-VAD-FMK. The effects of extracellular S1P are primarily mediated through S1P receptors (mainly S1PR-1 and −3), which are G protein-coupled receptors that activate the PI3K/Akt pathway. This activation inhibits caspase activation and, consequently, inhibits the execution of apoptosis in granulosa cells (Nakahara [39]). Z-VAD-FMK, which is cell-permeable, is known to inhibit the activity of caspase-3, −8 and −9 by binding to the catalytic sites of these caspases. Inhibition of these caspases prevents the execution of apoptosis (Men [38]). (DOCX 1386 kb)
