# Supplementary material for: Supplementation of transport and freezing media with anti-apoptotic drugs improves ovarian cortex survival
Source: J Ovarian Res. 2016 Feb 12;9:4. doi: 10.1186/s13048-016-0216-0 (PMC4751643; doi:10.1186/s13048-016-0216-0)
Supplement: Additional file 2: Figure S1. — Cellular anti-apoptotic mechanisms of S1P and Z-VAD-FMK. The effects of extracellular S1P are primarily mediated through S1P receptors (mainly S1PR-1 and −3), which are G protein-coupled receptors that activate the PI3K/Akt pathway. This activation inhibits caspase activation and, consequently, inhibits the execution of apoptosis in granulosa cells (Nakahara [39]). Z-VAD-FMK, which is cell-permeable, is known to inhibit the activity of caspase-3, −8 and −9 by binding to the catalytic sites of these caspases. Inhibition of these caspases prevents the execution of apoptosis (Men [38]). (DOCX 1386 kb) [file 13048_2016_216_MOESM2_ESM.docx]

**

***Additional Figure 1:*** *Cellular anti-apoptotic mechanisms of S1P and Z-VAD-FMK.*

*The effects of extracellular S1P are primarily mediated through S1P receptors (mainly S1PR-1 and -3), which are G protein-coupled receptors that activate the PI3K/Akt pathway. This activation inhibits caspase activation and, consequently, inhibits the execution of apoptosis in granulosa cells (Nakahara 2012).*

*Z-VAD-FMK, which is cell-permeable, is known to inhibit the activity of caspase-3, -8 and -9 by binding to the catalytic sites of these caspases. Inhibition of these caspases prevents the execution of apoptosis (Men 2006).*
